# Impact of clonal hematopoiesis of indeterminate potential on arterial atherothrombosis and venous thromboembolism: Protocol for a systematic review and meta-analysis

**DOI:** 10.1371/journal.pone.0328650

**Published:** 2025-07-16

**Authors:** Angela Todorovski, Tzu-Fei Wang, Evan Sterling, Erin Collins, Marc Carrier, Deborah Siegal, Natasha Kekre, Roy Khalifé, Yan Xu

**Affiliations:** 1 Department of Medicine, Ottawa Hospital Research Institute, University of Ottawa, Ottawa, Canada; 2 School of Epidemiology and Public Health, Faculty of Medicine, University of Ottawa, Ottawa, Canada; 3 University of Ottawa Library, Ottawa, Canada; Ataturk University Faculty of Medicine, TÜRKIYE

## Abstract

**Background:**

Clonal hematopoiesis of indeterminate potential (CHIP) is a novel risk factor for thromboembolic events. While CHIP is linked to an increased risk of incident atherothrombosis (ATE), the link between these two conditions varies across studies. Furthermore, the association between CHIP and incident venous thromboembolism (VTE) has not yet been well characterized. Among patients with established ATE and VTE, it is still unclear how CHIP carriership influences their health outcomes. We aim to conduct a systematic review and meta-analysis to: i) determine the impact of CHIP carriership on incident ATE and VTE; and ii) evaluate the prevalence and clinical consequences of CHIP mutations among individuals with established ATE or VTE.

**Methods:**

We will search MEDLINE, EMBASE, Scopus and CINAHL for randomized trials, cohort studies, or case control studies reporting thromboembolic events among adult CHIP carriers and non-carriers in two populations: i) individuals without prior ATE (coronary artery disease, myocardial infarction, ischemic stroke, peripheral arterial disease) or VTE (pulmonary embolism, deep vein thrombosis, superficial vein thrombosis); and ii) individuals with established ATE or VTE. Cross-sectional studies will be included to determine the prevalence of CHIP among individuals with established thromboembolic disease. The primary outcome will be incident ATE and VTE. Secondary outcomes will be: i) CHIP prevalence among individuals with established ATE or VTE; and ii) recurrent thromboembolism and treatment-associated bleeding among individuals with established ATE or VTE. We will use random-effects meta-analyses, with subgroup analyses by participant demographics, ATE and VTE risk factors, and CHIP-specific characteristics.

**Discussion:**

By understanding the prognostic impact of CHIP carriership, our findings will inform future research on CHIP’s role as a predictive biomarker for ATE and VTE in the general population and among individuals with established thromboembolic disease.

**Registration:**

This systematic review protocol was registered with the Internal Prospective Register of Systematic Reviews (PROSPERO, registration number CRD42024539923).

## 1. Introduction

One in four deaths worldwide are attributable to thrombosis, manifest as atherothrombosis (ATE, e.g., coronary artery disease, acute myocardial infarction, acute ischemic stroke, and peripheral artery disease) and venous thromboembolism (VTE, e.g., pulmonary embolism and deep vein thrombosis) [1]. Risks of ATE and VTE increase with age, and recent data have identified clonal hematopoiesis of indeterminate potential (CHIP), a set of age-dependent, inflammation-driven genetic changes in the hematopoietic system, as a novel predictor of ATE and VTE [2–4].

CHIP is defined as the presence of a somatic (acquired) mutation of a myeloid malignancy-associated gene with a variant allele fraction (VAF) of ≥2%, in the absence of a diagnosed hematologic disorder [5]. Over the past decade, CHIP has emerged as an important contributor to ATE. Using whole-exome sequencing data from two prospective cohorts that comprised 1,010 participants without a history of cardiovascular events, Jaiswal *et al* reported a 1.9-fold increased risk of ATE among CHIP carriers compared to their non-CHIP counterparts (odds ratio [OR] 1.9, 95% confidence interval [CI] 1.4 to 2.7) [2]. In the same report, a 4-fold increase in risk of early-onset myocardial infarction was observed among CHIP carriers compared to matched non-carriers among 7,245 participants who were less than 50 years of age at the time of their acute coronary syndrome [2].

An updated knowledge synthesis is needed for several reasons. First, while a systematic review and meta-analysis in 2022 reported a 1.6-fold increased risk of ATE among individuals with CHIP compared to those without CHIP (OR 1.6, 95% CI 1.3–2.1), more contemporary data with larger sample size (e.g., UK Biobank 500k cohort) have reported a much more modest impact of CHIP on ATE [6]. Second, increasing number of studies have now examined the impact of CHIP on VTE. For example, high prevalence of CHIP carriership has been observed among individuals with unprovoked pulmonary embolism [3], and the presence of CHIP has been associated with increased risks of incident VTE [7]. While the incidence of VTE is strongly correlated with increasing age [8], the impact of CHIP on VTE has not been summarized. Third, contemporary evidence indicates increased cardiovascular risk and mortality associated with CHIP detected far below 2% variant allele fraction (VAF) [9,10], a threshold that was originally established by detection limit associated with sequencing technologies [11]. There is heterogeneity in CHIP case identification due to differences in sequencing strategies (e.g., panel-based sequencing vs. whole genome sequencing), sequencing platforms (e.g., digital droplet polymerase chain reaction vs. next generation sequencing) and gene lists used for variant calling and curation. Finally, the prognostic impact of CHIP carriership on recurrent thromboembolic events and bleeding complications among adults with an established diagnosis of ATE or VTE has not been systematically reviewed.

## 2. Objectives

The primary objective of this systematic review and meta-analysis is to determine the impact of CHIP on the incidence of ATE and VTE among individuals with no previous history of thromboembolic disease. Secondary objectives are to: 1) determine the prevalence of CHIP among patients with first episode of ATE and VTE with stratification by their respective risk factors; and 2) evaluate the impact of CHIP on recurrent thromboembolism and treatment-related bleeding complications among patients with established ATE and VTE.

## 3. Methods

This protocol is reported in accordance with the Preferred Reporting Items for Systematic Review and Meta-Analysis Protocols (PRISMA-P) Statement (2015) [12]. Amendments to the current protocol, if required, will be reported in the final study report.

### Eligibility criteria

#### Study design and characteristics,.

For the primary objective, we will include randomized controlled trials, prospective and retrospective cohort studies, and case-control studies. For the secondary objective, we will include aforementioned study types with addition of cross-sectional studies to capture CHIP prevalence.

We will include peer-reviewed published manuscripts. We will exclude case reports, case series, pre-prints, trial registries, protocols, or unpublished or non-peer reviewed studies, including grey literature. These exclusions are intended to ensure that each screened study has appropriate peer-review. The findings of relevant conference abstracts will only be included if a subsequent targeted search identifies a peer-reviewed manuscript of the same study at the time of data extraction. There will be no restriction based on the year, language, or geographical region. Non-English manuscripts will be translated using an online translation service [13].

#### Participants.

**Fig 1:**
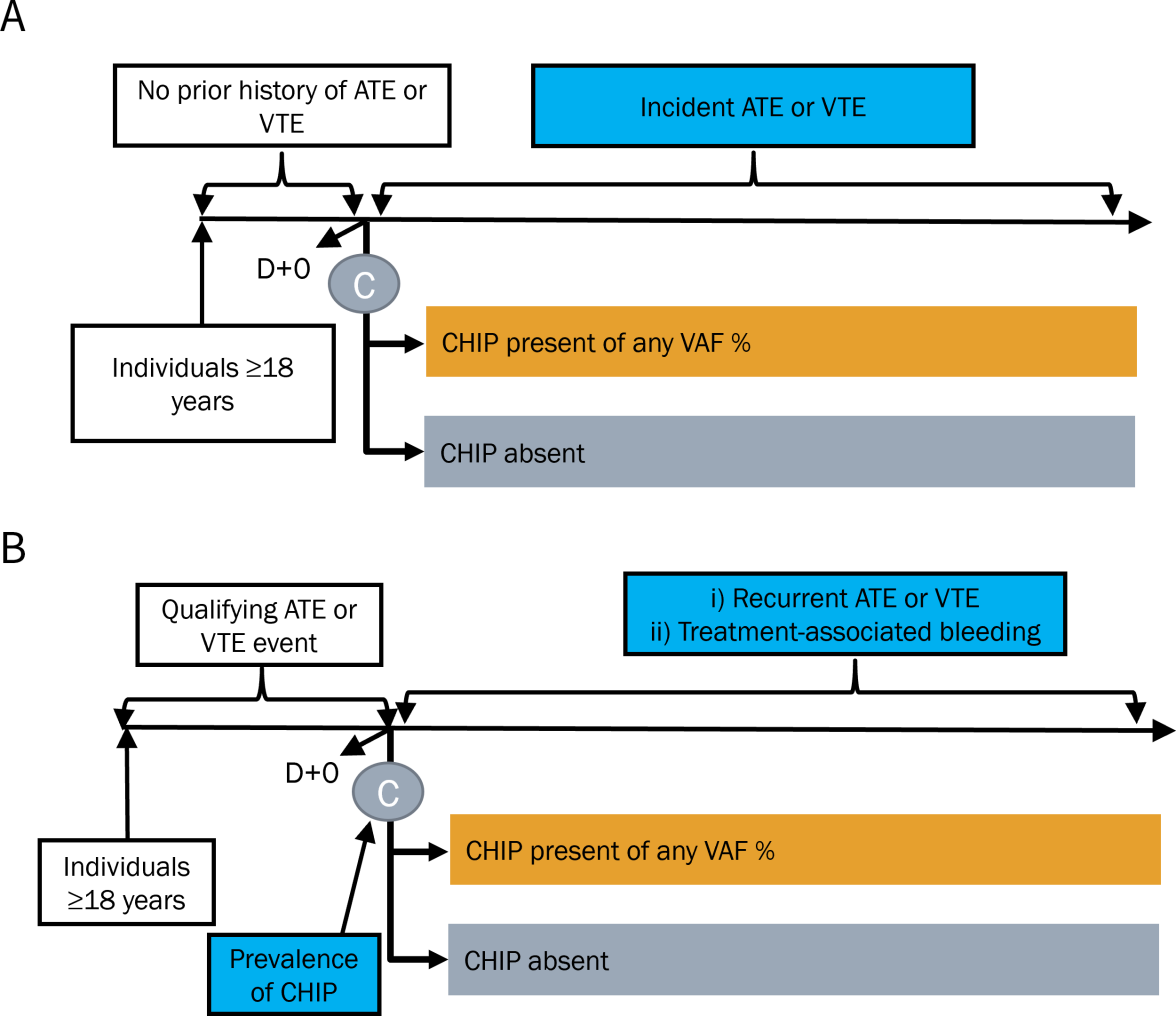
Flow diagram for eligible studies evaluating populations without prior atherothrombosis or venous thromboembolism (A) and populations with established atherothrombosis and venous thromboembolism (B) of this systematic review and meta-analysis. ATE, atherothrombosis; VTE, venous thromboembolism, CHIP, clonal hematopoiesis of indeterminate potential; VAF, variant allele fraction.

To determine the impact of CHIP on incident ATE or VTE, we will include studies that enrolled individuals aged 18 years of age or older without a prior history of ATE or VTE at the time of CHIP testing (**Fig 1A**).

To determine the prevalence and prognostic impact of CHIP among those with history of thromboembolism, we will include studies of patients 18 years of age or older with an established diagnosis of ATE or VTE at the time of their CHIP testing (**Fig 1B**). ATE includes one or more of the following: coronary artery disease, acute myocardial infarction, acute ischemic stroke, or peripheral arterial disease (as defined in individual studies). Similarly, VTE will be defined by one or more of the following: pulmonary embolism, deep vein thrombosis, or superficial vein thrombosis (as defined in individual studies). We will include VTE involving atypical sites, such as cerebral venous thrombosis and splanchnic vein thrombosis. Study definitions of qualifying ATE or VTE diagnoses will be recorded.

We will include individuals of any sex, gender, racial, ethnic and ancestry groups, and geographical location. We will not restrict patients based on use of antithrombotic or conditions associated with CHIP (e.g., cancer or receipt of anti-cancer therapy), and these characteristics will be included will be recorded if available. Consistent with the definition of CHIP, individuals with an established hematologic diagnosis (e.g., myelodysplastic syndrome, acute myeloid leukemia, myeloproliferative neoplasms) will be excluded.

Studies involving individuals <18 years of age at time of enrolment will be excluded, as CHIP is an age-related condition that is very rare to occur in the pediatric population [14].

#### Exposure/Comparators.

The exposure of interest is CHIP carriership. While CHIP is defined as variant allele frequency (VAF) ≥2%, we will not restrict by VAF in this systematic review and meta-analysis, based on emerging data that suggest increased thromboembolic risks associated with CHIP carriers with VAF < 2% [15–17]. The comparator group will be patients without CHIP carriership at any detectable VAF.

#### Outcomes.

The primary outcome will be ATE or VTE. ATE will include incident diagnosis of coronary artery disease, acute myocardial infarction, acute ischemic stroke, or peripheral artery disease [18]. Coronary artery disease will be defined by the 2023 AHA/ACC guideline, and includes individuals with angina symptoms (with or without imaging confirmation), those following acute myocardial infarction or after coronary revascularization procedure, and those with ischemic cardiomyopathy [19]. Acute myocardial infarction will be defined as per the 2018 Joint Task Force universal definition of MI, and will be inclusive of non-ST elevation myocardial infarction or ST elevation myocardial infarction [20]. Acute ischemic stroke will be defined as any neurological dysfunction caused by focal cerebral, spinal, or retinal infarction [21]. Peripheral artery disease will include ATE involving the upper or lower extremities, as well as cervical arteries (e.g., carotid artery stenosis).

VTE will be defined as imaging-confirmed diagnosis of pulmonary embolism, upper and lower extremity deep vein thrombosis, superficial vein thrombosis, or VTEs involving atypical sites, including cerebral venous thrombosis and splanchnic vein thrombosis. We will include incidental VTE and VTE of any clot burden, including subsegmental pulmonary embolism and isolated distal deep vein thrombosis. Studies that reported VTE outcomes without appropriate imaging will not be included.

If an included study did not specify the definition used to classify the outcome, diagnostic criteria as reported by the study will be used as the endpoint of interest and recorded.

As part of the secondary objective (**Fig 1B**), we will include studies that captured treatment-related bleeding complications as defined by the individual studies.

### Information sources and search strategy

In collaboration with a research librarian (E.S.), a comprehensive systematic literature search will be performed using Medline, Embase, Scopus, and CINAHL from database inception to July 2024. A draft Medline search strategy is included in S1 Appendix and will be adapted across the other databases. We will use Medical Subject Heading (MeSH) terms with supplementation by keywords, with vocabulary and syntax adjusted based on the databases queried. The search has been designed with two concept groups: 1) Clonal hematopoiesis or genetic markers associated with CHIP, and 2) cardiovascular disease/ATE and VTE.

### Data management

Search results from the included databases will be uploaded into Covidence ® (Alfred Hospital, Melbourne, Australia), and duplicate references will be excluded. Ten articles for level 1 (title and abstract) screening, and ten articles for level 2 (full text review) screening will be piloted by all study authors to ensure agreement in handling of inclusion/exclusion criteria. Studies with multiple publications (e.g., interim analyses, extended follow up publications) will be assessed as a single study by cross referencing trial name and registration, author name, and institution.

### Selection process

At least two independent reviewers will use Covidence to screen titles and abstracts based on the pre-selected inclusion and exclusion criteria. At both level 1 (title and abstract) and level 2 (full text review) screening, each title, abstract, and eligible full text article will be assessed by two reviewers. Disagreements in screening will be resolved initially by discussion between the two reviewers, and eventually by a third and adjudicating reviewer if the conflict remains unresolved. Articles excluded at title and abstract screening and full text review will be recorded and presented in a PRISMA flow diagram. At full text review, reasons for exclusion will be selected by the reviewers and will be recorded in the flow chart. Study authors will not be blinded to the study title and author information. Additional notes or tags that are added by reviewers to the articles through the Covidence platform are also not blinded.

### Data collection

A standardized data extraction form will be developed and piloted in Microsoft Excel to record author, publication year, study design, follow-up duration, number of patients (total and per intervention group) included, patient demographics, baseline ATE and VTE risk factors, CHIP detection methods, CHIP-related risk factors, and outcomes. Full list of data extracted are included in **Table 1**. Where an eligible study has multiple publications involving the same cohort of patients, outcomes for all studies will be collected in a single collection form. Data will be extracted by two authors independently, and each study will be extracted in duplicate. All reviewers will pilot extraction data for one study. Any discrepant data fields will be resolved by consensus, with a third and adjudicating reviewer to resolve any conflicts that remain.

**Table 1:** Data to be extracted from included studies.

Study characteristics	Study ID, author, publication year, study design, follow-up duration, duration of follow-up, and geography
**Participant characteristics**	Number of participants included, mean/median age, % female, race, ethnicity or ancestry, use of antiplatelets or anticoagulants
**Risk factors for ATE**	History of ATE (coronary artery disease, myocardial infarction, ischemic stroke, peripheral artery disease), smoking status, hyperlipidemia, obesity, smoking, hypertension
**Risk factors for VTE**	Active malignancy, use of exogenous estrogen, surgery <90 days, immobilization <90 days, family history
**CHIP detection method**	Sequencing strategy (targeted vs. whole genome/exome), sequencing platform, covered gene list
**Exposure (CHIP) characteristics**	Mutation type, mean/median VAF, % with high-burden CHIP (defined as ≥10% VAF), VAF threshold used to define CHIP carriership
**Outcomes**	Number of participants with ATE and its subtypes (coronary artery disease, myocardial infarction, ischemic stroke, peripheral artery disease)
**Covariate assessment**	Matching technique used (regression, propensity score matching, historical controls), matching variables used

CHIP, clonal hematopoiesis of indeterminate potential; ATE, atherothrombotic event; VTE, venous thromboembolism; VAF, variant allele frequency.

### Risk of bias in individual studies

Risk of bias assessment will be conducted by two reviewers independently using the QUIPS tool, which is used to evaluate the prognosis studies [22]. Studies will be analyzed based on five bias domains (S2 Appendix): study participation, study attrition, prognostic factor measurement, outcome measurements, study confounding, and statistical analysis and reporting. Each of the categories will be scored using the following four descriptions: yes (low risk of bias), probably yes, probably no, and definitely no (high risk of bias). A high risk of bias is assigned if a study contains ≥1 domains as “definitely no”, or ≥2 domains marked as “definitely no” or probably no”. A low risk of bias is assigned if all domains are assessed as “probably yes” or “definitely yes.” All other studies will be considered as unclear risk of bias. Disagreements will be resolved by discussion between the two reviewers, with involvement of a third reviewer if necessary.

### Data synthesis

To determine the association between CHIP and occurrence of ATE or VTE, we will derive pooled ORs with 95% CIs at the study level. Additionally, we will pool the incidence/ recurrence rates (reported as events per person-years) and rate ratios using data from randomized controlled trials, prospective cohort and retrospective cohort studies. Each arm of a randomized trial will be treated as a prospective cohort for this step. We will generate pooled summary statistics using logit transformation of proportions, followed by inverse-variance weighted random effects meta-analysis using the DerSimonian and Laird model for CHIP carriers and non-carriers [23]. If there are insufficient and heterogenous data to perform meta-analysis at the subgroup level, a narrative synthesis will be done. A descriptive summary of the key characteristics of each of the included studies will be tabulated.

To determine the prevalence of CHIP carriership, we will analyze individuals with CHIP mutations as proportion of all ATE and VTE patients at each study level. This will be followed by transformation of proportions and random-effects meta-analysis using the DerSimonian and Laird method detailed above. Heterogeneity between studies will be assessed using visual inspection and the I² statistic. We plan to perform complete case analysis as primary analysis while capturing the proportion of missing primary outcomes of interest. When missingness of a specific outcome is ≥ 5% among included studies, we will perform four assumptions as sensitivity analyses: best case scenario, none of the participants with missing data had the outcome, all participants with missing data had the outcome, and worse case scenario.

We will conduct subgroup analyses to assess the robustness of the association between CHIP and thromboembolic outcomes by the following potential variables provided sufficient data are available: age, sex, self-reported race, ethnicity and ancestry, geographical location of the reported study, high-risk CHIP as defined by VAF ≥ 10%, specific CHIP-defining genes (e.g., *DNMT3A*, *TET2*, *ASXL1, JAK2*), presence of hyperlipidemia, obesity, smoking, hypertension, VTE associated with a persistent risk factor (e.g., active malignancy, autoimmune conditions), VTE associated with a transient risk factors (e.g., surgery, immobilization, acute medical illness, COVID-19 infections), VTE associated with no observed risk factor (e.g., unprovoked VTE), and presence of family history. Interaction testing will be performed to determine subgroup effects, and its associated Chi-squared statistic and *p*-value will be reported.

We will perform sensitivity analyses by excluding studies with high risk of bias using the QUIPS tool, and studies using adjudicated ATE and VTE endpoints rather than those reported in routine clinical care (e.g., administrative database coding). If at least ten studies are extracted in the primary analysis, publication bias will be visually assessed using funnel plots and through Egger’s test.

Finally and data permitting, we will assess the strength of the body of evidence using the Grades of Recommendation, Assessment, Development and Evaluation (GRADE) approach [24]. The QUIPS tool will be incorporated into the GRADE process as part of rating for risk of bias.

### Dissemination

This systematic review and meta-analysis will use publicly available information; as such, research ethics approval will not be required. While handling of individual patient-level data are not expected in context of this study-level systematic review and meta-analysis, we will seek appropriate ethics approval and data sharing agreements if individual patient-level data are required. Search strategy, extraction protocols, and handled discrepancies will be reported using the Preferred Reporting Items for Systematic Reviews and Meta-Analyses 2020 guidelines [25]. We aim to submit this work for presentation at an international conference and submit our results for publication in a peer-reviewed journal. Extracted data used for data synthesis will be available to researchers upon request to the corresponding author, upon completion and publication of this systematic review and meta-analysis.

## 4. Conclusions

Despite the evolving role of CHIP as a novel cardiovascular biomarker, the prognostic impact of CHIP for incident ATE and VTE remain uncertain. Furthermore, the clinical consequences of carrying CHIP mutations among those with established ATE and VTE remain poorly understood. Our systematic review and meta-analysis will establish summary estimates on CHIP’s performance as a prognostic tool, thereby informing the net clinical benefit associated with CHIP screening in the general population and individuals with established cardiovascular disease. This is especially relevant given the lack of consensus recommendations on CHIP screening [26]. In addition, no interventional study has prospectively incorporated CHIP to evaluate its performance as a predictive variable to inform precision prevention of cardiovascular disease despite promising post-hoc data [27]. Therefore, our study outputs will also inform both primary and secondary prevention strategies that incorporate CHIP in adequately powered interventional trials. By understanding the intersection between ATE, VTE and CHIP, our research output will improve the understanding of this thrombo-inflammatory nexus and catalyze innovative research aimed at reducing the global burden of ATE and VTE through CHIP-directed therapies.

## Supporting information

S1 Appendix Medline search strategy. (DOCX)

S2 Appendix Components of the Quality in Prognosis Studies (QUIPS) tool for risk of bias assessment. (DOCX)

S1 File PRISMA-P-checklist. (DOCX)

## Abbreviations

CHIP: Clonal hematopoiesis of indeterminate potential
CINAHL: Cumulated Index to Nursing and Allied Health Literature
PROSPERO: International Prospective Register of Systematic Reviews
ATE: Atherothrombotic events
VTE: Venous thromboembolism

## References

[1] Goldhaber SZ, Bounameaux H. Pulmonary embolism and deep vein thrombosis. Lancet. 2012;379(9828):1835–46. doi: 10.1016/S0140-6736(11)61904-1 22494827

[2] Jaiswal S, Natarajan P, Silver AJ, Gibson CJ, Bick AG, Shvartz E, et al. Clonal hematopoiesis and risk of atherosclerotic cardiovascular disease. N Engl J Med. 2017;377:111–21. 28636844 10.1056/NEJMoa1701719 PMC6717509

[3] Soudet S, Jedraszak G, Evrard O, Marolleau JP, Garcon L, Pietri MAS. Is hematopoietic clonality of indetermined potential a risk factor for pulmonary embolism? TH Open. 2021;5(3):e338–42. doi: 10.1055/s-0041-1733856 34414354 PMC8370792

[4] Zon B, Sekar A, Clapham K, Niroula A, Bick AG, Gibson CJ, et al. Clonal Hematopoiesis and venous thromboembolism in the UK Biobank. Blood. 2023;142(Supplement 1):568–568. doi: 10.1182/blood-2023-180764

[5] Marnell CS, Bick A, Natarajan P. Clonal hematopoiesis of indeterminate potential (CHIP): linking somatic mutations, hematopoiesis, chronic inflammation and cardiovascular disease. J Mol Cell Cardiol. 2021;161:98–105. doi: 10.1016/j.yjmcc.2021.07.004 34298011 PMC8629838

[6] Kessler MD, Damask A, O’Keeffe S, Banerjee N, Li D, Watanabe K, et al. Common and rare variant associations with clonal haematopoiesis phenotypes. Nature. 2022;612(7939):301–9. doi: 10.1038/s41586-022-05448-9 36450978 PMC9713173

[7] Zon RL, Sekar A, Clapham K, Oren O, Niroula A, Bick AG, et al. JAK2-mutant clonal hematopoiesis is associated with venous thromboembolism. Blood. 2024;144(20):2149–54. doi: 10.1182/blood.2024024187 39102652 PMC11600088

[8] White RH. The epidemiology of venous thromboembolism. Circulation. 2003;107(23 Suppl 1):I4-8. doi: 10.1161/01.CIR.0000078468.11849.66 12814979

[9] Wang S, Hu S, Luo X, Bao X, Li J, Liu M, et al. Prevalence and prognostic significance of DNMT3A- and TET2- clonal haematopoiesis-driver mutations in patients presenting with ST-segment elevation myocardial infarction. EBioMedicine. 2022;78:103964. doi: 10.1016/j.ebiom.2022.103964 35339897 PMC8960977

[10] Arends CM, Liman TG, Strzelecka PM, Kufner A, Lowe P, Huo S, et al. Associations of clonal hematopoiesis with recurrent vascular events and death in patients with incident ischemic stroke. Blood. 2023;141:787–99. 36441964 10.1182/blood.2022017661

[11] Todorovski A, Wang T-F, Carrier M, Xu Y. CHIP away at the marrow-clot connection: inflammation, clonal hematopoiesis, and thromboembolic disease. Blood Adv. 2025;9(2):343–53. doi: 10.1182/bloodadvances.2024014430 39561373 PMC11787476

[12] Moher D, Shamseer L, Clarke M, Ghersi D, Liberati A, Petticrew M, et al. Preferred reporting items for systematic review and meta-analysis protocols (PRISMA-P) 2015 statement. Syst Rev. 2015;4(1):1. doi: 10.1186/2046-4053-4-1 25554246 PMC4320440

[13] Rockliffe L. Including non‐English language articles in systematic reviews: a reflection on processes for identifying low‐cost sources of translation support. Res Synth Methods. 2022;13:2–5. 34169665 10.1002/jrsm.1508

[14] Jaiswal S, Ebert BL. Clonal hematopoiesis in human aging and disease. Science. 2019;366(6465):eaan4673. doi: 10.1126/science.aan4673 31672865 PMC8050831

[15] Vlasschaert C, Mack T, Heimlich JB, Niroula A, Uddin MM, Weinstock JS. A practical approach to curate clonal hematopoiesis of indeterminate potential in human genetic datasets. Blood. 2023. doi: blood.2022018825 10.1182/blood.2022018825 PMC10273159 36652671

[16] Fuster JJ. Clonal hematopoiesis and coronary artery disease-a deep connection. JAMA Cardiol. 2024;9(3):242–4. doi: 10.1001/jamacardio.2023.5106 38198161

[17] Zhao K, Shen X, Liu H, Lin Z, Li J, Chen S, et al. Somatic and germline variants and coronary heart disease in a Chinese population. JAMA Cardiol. 2024;9(3):233–42. doi: 10.1001/jamacardio.2023.5095 38198131 PMC10782380

[18] Khorana AA, McNamara MG, Kakkar AK, Streiff MB, Riess H, Vijapurkar U, et al. Assessing full benefit of rivaroxaban prophylaxis in high-risk ambulatory patients with cancer: thromboembolic events in the randomized CASSINI trial. TH Open. 2020;4(2):e107–12. doi: 10.1055/s-0040-1712143 32462111 PMC7245534

[19] Writing Committee Members, Virani SS, Newby LK, Arnold SV, Bittner V, Brewer LC, et al. 2023 AHA/ACC/ACCP/ASPC/NLA/PCNA Guideline for the Management of Patients With Chronic Coronary Disease: A Report of the American Heart Association/American College of Cardiology Joint Committee on Clinical Practice Guidelines. J Am Coll Cardiol. 2023;82:833–955. 10.1016/j.jacc.2023.04.003 37480922

[20] Thygesen K, Alpert JS, Jaffe AS, Chaitman BR, Bax JJ, Morrow DA, et al. Fourth Universal Definition of Myocardial Infarction (2018). Circulation. 2018;138(20):e618–51. doi: 10.1161/CIR.0000000000000617 30571511

[21] Sacco RL, Kasner SE, Broderick JP, Caplan LR, Connors JJB, Culebras A, et al. An updated definition of stroke for the 21st century: a statement for healthcare professionals from the American Heart Association/American Stroke Association. Stroke. 2013;44(7):2064–89. doi: 10.1161/STR.0b013e318296aeca 23652265 PMC11078537

[22] Hayden JA, van der Windt DA, Cartwright JL, Côté P, Bombardier C. Assessing bias in studies of prognostic factors. Ann Intern Med. 2013;158(4):280–6. doi: 10.7326/0003-4819-158-4-201302190-00009 23420236

[23] DerSimonian R, Laird N. Meta-analysis in clinical trials. Control Clin Trials. 1986;7(3):177–88. doi: 10.1016/0197-2456(86)90046-2 3802833

[24] Schünemann HBJ Guyatt G, Oxman A, editors. GRADE handbook for grading quality of evidence and strength of recommendations. [Internet]. 2013. Available from: guidelinedevelopment.org/handbook

[25] Page MJ, McKenzie JE, Bossuyt PM, Boutron I, Hoffmann TC, Mulrow CD, et al. The PRISMA 2020 statement: an updated guideline for reporting systematic reviews. BMJ. 2021;372:n71. doi: 10.1136/bmj.n71 33782057 PMC8005924

[26] Libby P, Sidlow R, Lin AE, Gupta D, Jones LW, Moslehi J, et al. Clonal hematopoiesis: crossroads of aging, cardiovascular disease, and cancer: JACC review topic of the week. J Am Coll Cardiol. 2019;74:567–77. 31345432 10.1016/j.jacc.2019.06.007 PMC6681657

[27] Svensson EC, Madar A, Campbell CD, He Y, Sultan M, Healey ML, et al. TET2-Driven clonal hematopoiesis and response to canakinumab: an exploratory analysis of the CANTOS randomized clinical trial. JAMA Cardiology. 2022;7:521–8. 35385050 10.1001/jamacardio.2022.0386 PMC8988022

