# Supplementary material for: Impact of clonal hematopoiesis of indeterminate potential on arterial atherothrombosis and venous thromboembolism: Protocol for a systematic review and meta-analysis
Source: PLoS One. 2025 Jul 16;20(7):e0328650. doi: 10.1371/journal.pone.0328650 (PMC12266447; doi:10.1371/journal.pone.0328650)
Supplement: S1 Appendix — (DOCX) [file pone.0328650.s001.docx]

**S1 Appendix –** Medline search strategy

| 1 | Clonal Hematopoiesis/ |
| --- | --- |
| 2 | (Clonal* adj4 hematopoi*).ti,ab,kf. |
| 3 | ((Clonal* or idiopathic) adj3 cytopeni*).ti,ab,kf. |
| 4 | or/1-3 |
| 5 | DNA Methyltransferase 3A/ or Janus Kinase 2/ or Genes, p53/ |
| 6 | (ASXL1* or ASXL 1* or DNMT3A* or DNMT 3A* or JAK2* or JAK 2* or PPM1D* or PPM 1D* or SF3B1* or SRSF2* or TET2* or TET 2* or TP53* or TP 53*).ti,ab,kf,nm. |
| 7 | or/1-6 |
| 8 | cardiovascular diseases/ or heart diseases/ or exp myocardial ischemia/ or vascular diseases/ or stroke/ or exp ischemic stroke/ or "embolism and thrombosis"/ or pulmonary embolism/ or venous thromboembolism/ or exp venous thrombosis/ or peripheral vascular diseases/ or exp peripheral arterial disease/ or exp thrombophlebitis/ |
| 9 | (cardiolog* or cardiovasc* or cardiac* or heart* or coronary or infarct* or STEMI or NSTEMI or (angina adj2 (unstable or preinfarction or rest)) or vascular* or arter* or athero* or vein* or venous* or stroke).ti,ab,kf. |
| 10 | (thrombos?s or thrombus or thrombi or thromboemb* or Thrombophlebit* or ((Pulmonary or lung or vein or venous) adj2 emboli*) or clot* or VTE or DVT or ((limb or peripher*) adj2 ischemia)).ti,ab,kf. |
| 11 | or/8-10 |
| 12 | 7 and 11 |
| 13 | 12 and (review or case reports or comment or editorial).pt. |
| 14 | 12 not 13 |
| 15 | exp animals/ or exp animal experimentation/ or exp models animal/ |
| 16 | exp humans/ or exp human experimentation/ |
| 17 | 14 not (15 not 16) |

**No language or date restriction has been applied.*
