# Supplementary material for: Impact of clonal hematopoiesis of indeterminate potential on arterial atherothrombosis and venous thromboembolism: Protocol for a systematic review and meta-analysis
Source: PLoS One. 2025 Jul 16;20(7):e0328650. doi: 10.1371/journal.pone.0328650 (PMC12266447; doi:10.1371/journal.pone.0328650)
Supplement: S2 Appendix — (DOCX) [file pone.0328650.s002.docx]

**S2 Appendix -** Components of the Quality in Prognosis Studies (QUIPS) tool for risk of bias assessment

| **Bias Domain** | **Optimal Characteristics** | **Prompting Items and Considerations** | **Risk of Bias Ratings** |
| --- | --- | --- | --- |
| **1. Study Participation** | Study sample represents population of interest | a. Adequate participation of eligible persons  b. Description of source population  c. Baseline study sample described  d. Sampling frame and recruitment described  e. Recruitment period and location described  f. Inclusion/exclusion criteria described | **High**: Likely difference between participants and nonparticipants.  **Moderate**: Possible difference.  **Low**: Unlikely difference. |
| **2. Study Attrition** | Follow-up participants represent study sample | a. Adequate response rate  b. Attempt to collect info on dropouts  c. Reasons for loss to follow-up described  d. Description of lost participants  e. No key differences between completers and dropouts | **High**: Likely difference between completers and non-completers.  **Moderate**: Possible difference.  **Low**: Unlikely difference. |
| **3. Prognostic Factor Measurement** | Prognostic factor measured similarly, validly, reliably | a. Clear definition of Prognostic factor b. Valid and reliable measurement  c. Continuous variables or appropriate cut points used  d. Same measurement method and setting for all  e. High data completeness  f. Appropriate imputation used for missing prognostic factor data | **High**: Likely differential measurement.  **Moderate**: Possible differential measurement.  **Low**: Unlikely differential measurement. |
| **4. Outcome Measurement** | Outcome measured similarly, validly, reliably | a. Clear definition of outcome  b. Valid and reliable measurement  c. Same method and setting for all  d. No differential measurement by prognostic factor level | **High**: Likely outcome measurement differs by prognostic factor level.  **Moderate**: Possible difference.  **Low**: Unlikely difference. |
| **5. Study Confounding** | Confounders appropriately measured and accounted for | a. All important confounders measured  b. Confounders clearly defined  c. Valid and reliable measurement of confounders  d. Same setting and method for all participants  e. Appropriate imputation for missing confounder data  f. Confounding addressed in design  g. Confounding addressed in analysis | **High**: Likely distortion of prognostic factor -outcome relation.  **Moderate**: Possible distortion.  **Low**: Unlikely distortion. |
| **6. Statistical Analysis and Reporting** | Appropriate analysis; complete reporting | a. Adequate data presentation  b. Model building strategy appropriate  c. Statistical model appropriate for study design  d. No selective reporting | **High**: Likely spurious or biased results.  **Moderate**: Possible bias.  **Low**: Unlikely bias. |
